# An alternative analgesic agent for intra-articular corticosteroid injections (IAI) in juvenile idiopathic arthritis (JIA) – a fentanyl lollipop

**DOI:** 10.1186/1546-0096-9-S1-P205

**Published:** 2011-09-14

**Authors:** Z Zareen, D Finn, O Killeen

**Affiliations:** 1 National Centre for Paediatric Rheumatology, Our Lays Children Hospital, Crumlin, Dublin, Ireland

## Background

Intra articular injections are an effective and widely used technique that involes the direct application of a corticosteroid compound such as triamcinalone hexacetonide into a joint with active synovitis. Local IAIs can avoid many of the unwanted adverse effects of sytemic steroids but in children younger than 11 years or those with a polyarticular presentation a general anaesthetic is typically required. Traditional local anaesthetic agents include topical agents such as lignocaine or gaseous agents such as entonox (nitrous oxide). We have introduced an unique form of analgesia for this procedure since the establishment of our national unit in 2006 in form of fentanyl lollipops (Actiq).

## Aim

To evaluate the efficacy & safety of fentanyl lollipops for children with JIA undergoing IAIs.

## Methods

A total of 19 patients were studied . Pain scores an hour after the procedure were recorded using a 0–10 cm visual analogue scale (VAS). Parents and patients were reinterviewed at a later date regarding the effectiveness of the lollipops as a form of pain relief and whether they would consider this form of analgesia for their child again.

## Results

A total of 25 joints were injected in 19 patients (median age14.6 yrs, range 10.4-16.6yrs). The median patient and parent pain scores were 1.5 and 4.0 respectively. There were no serious adverse events in any patient. All parents agreed that fentanyl lollipops were safe and effective, and over 90% would opt for this form of analgesia again.

## Conclusion

Fentanyl lollipops are a safe and an effective form of analgesia for intra-articular injections in children. A significant number of parents overestimated pain related to the procedure compared to their child.

